# Ostreolysin A/Pleurotolysin B and Equinatoxins: Structure, Function and Pathophysiological Effects of These Pore-Forming Proteins

**DOI:** 10.3390/toxins9040128

**Published:** 2017-04-05

**Authors:** Robert Frangež, Dušan Šuput, Jordi Molgó, Evelyne Benoit

**Affiliations:** 1 Institute of Preclinical Sciences, Veterinary Faculty, University of Ljubljana; 1115-Ljubljana, Slovenia; Robert.Frangez@vf.uni-lj.si; 2 Laboratory for Cell Physiology and Toxinology, Institute of Pathophysiology, School of Medicine, University of Ljubljana, P.O. Box 11, 1105-Ljubljana, Slovenia; dusan.suput@mf.uni-lj.si; 3 DRF/Institut de Sciences de la Vie Frédéric Joliot/SIMOPRO, CEA de Saclay, and Institut des Neurosciences Paris-Saclay (Neuro-PSI), UMR 9197 CNRS/Université Paris-Sud, 91190 Gif-sur-Yvette, France; jordi.molgo@cea.fr

**Keywords:** ostreolysin A/pleurotolysin B, equinatoxins, pore-forming proteins, biological effects

## Abstract

Acidic ostreolysin A/pleurotolysin B (OlyA/PlyB, formerly known as ostreolysin (Oly), and basic 20 kDa equinatoxins (EqTs) are cytolytic proteins isolated from the edible mushroom *Pleurotus ostreatus* and the sea anemone *Actinia equina*, respectively. Both toxins, although from different sources, share many similar biological activities: (i) colloid-osmotic shock by forming pores in cellular and artificial membranes enriched in cholesterol and sphingomyelin; (ii) increased vascular endothelial wall permeability in vivo and perivascular oedema; (iii) dose-dependent contraction of coronary vessels; (iv) haemolysis with pronounced hyperkalaemia in vivo; (v) bradycardia, myocardial ischemia and ventricular extrasystoles accompanied by progressive fall of arterial blood pressure and respiratory arrest in rodents. Both types of toxins are haemolytic within nanomolar range concentrations, and it seems that hyperkalaemia plays an important role in toxin cardiotoxicity. However, it was observed that the haemolytically more active EqT III is less toxic than EqT I, the most toxic and least haemolytic EqT. In mice, EqT II is more than 30 times more toxic than OlyA/PlyB when applied intravenously. These observations imply that haemolysis with hyperkalaemia is not the sole cause of the lethal activity of both toxins. Additional mechanisms responsible for lethal action of the two toxins are direct effects on heart, coronary vasoconstriction and related myocardial hypoxia. In this review, we appraise the pathophysiological mechanisms related to the chemical structure of OlyA/PlyB and EqTs, as well as their toxicity.

## 1. Introduction

Ostreolysin (Oly) and equinatoxins (EqTs) belong to the group of pore-forming proteins with a defined native conformation which, upon an environmental trigger, is changed spontaneously to enable formation of a transmembrane pore consisting of several protein monomers [1]. Recently, Oly was found to consist of two proteins, ostreolysin A (OlyA) and pleurotolysin B (PlyB), produced by the edible mushroom *Pleurotus ostreatus* in a 9:1 molar ratio, respectively, and with respective ~15 and ~59 kDa molecular masses [2]. Both OlyA/PlyB and EqT II are characterized structurally by a dominant β-structure scaffold that keeps a constant global domain tertiary structure, while the polypeptide elements needed for membrane penetration are provided by minor local conformational changes [3,4]. Like other pore-forming toxins, both Oly and EqTs bind to the cell membrane by recognising a specific membrane component. EqT II recognises the sphingomyelin head group [5], while Oly specifically binds to membrane domains containing both sphingomyelin and cholesterol [2,6]. This binding results in protein oligomerization at the membrane surface, and the formation of transmembrane pores, leading to colloid-osmotic mechanisms resulting in cell lysis. The aim of this review is to summarise the pathophysiological mechanisms related to the chemical structure of both proteins, and to discuss their toxicity on various levels of biological organization.

## 2. Ostreolysin A/Pleurotolysin B

### 2.1. Origin

Oly was reported to be an acidic, cytolytic 15 kDa protein that was first isolated from the edible oyster mushroom *Pleurotus ostreatus* [7]. This protein was shown to be highly similar to Aa-Pri1, a predicted protein from *Agrocybe aegerita* [8] and similar to Asp-haemolysin from *Aspergillus fumigatus* [9] and the non-haemolytic proteins P16 and P18 from the bacterium *Clostridium bifermentans* [10]. Recently, Oly was reported to consist of two proteins, ostreolysin A (OlyA) and pleurotolysin B (PlyB) with membrane-attack complex/perforin (MACPF) domain, produced by the edible mushroom *Pleurotus ostreatus* in a respective 9:1 molar ratio and with molecular masses of ~15 and ~59 kDa, respectively [2,11]. Electron microscopy studies revealed that these two proteins, assuming a 1:1 ratio, form a subunit of a predominantly 13-meric rosette-like structure with a central hydrophilic pore of 4.9 nm inner diameter which is non-selectively permeable to ions and smaller neutral solutes [2]. Although a colloid-osmotic type mechanism responsible for the OlyA/PlyB cytolytic effect has been proposed, the location of molecules on lipid membranes and their ratio in the pore complex remain uncertain [11]. However, more recently, combined crystallography and cryo-electron microscopy were used to define the atomic details of the complex formed by pleurotolysin A (PlyA), which is very similar to OlyA, and PlyB. The results showed that each subunit of the 13-fold pore of 8 nm in inner diameter comprised a PlyB molecule atop a PlyA dimer, leading thus to a respective 2:1 ratio between PlyA and PlyB [12].

### 2.2. Chemical Structure and Biological Properties

OlyA/PlyB is a pore-forming cytolysin which belongs to the larger group of highly homologous proteins called aegerolysins (Pfam PF06355, InterPro IPR009413), currently comprising more than 350 entries in the NCBI databases. The structure, putative functional characteristics and occurrence of aegerolysins across the tree of life have been reviewed in details elsewhere [13,14]. OlyA/PlyB is specifically expressed during the formation of mushroom fruit bodies, but its primary role in the producing organism is not yet clarified [7,15]. The complete structure of OlyA/PlyB complex is not yet solved. However, the protein characterisation by means of circular dichroism, UV-absorption and fluorescence spectroscopy revealed that, under physiological conditions, each of the molecules adopts a monomeric and thermodynamically stable native-like conformation characterized by rigid tertiary and predominantly β-sheet secondary structures. This compact native state is necessary for the protein binding to cholesterol/sphingomyelin membrane domains [4]. Recently, the transition from PlyA and PlyB monomeric forms into oligomeric membrane prepores and then into pores was detailed using a variety of structural and biophysical approaches, gaining further insight into the structures of not only the lipid binding PlyA protein and the pore-forming MACPF component PlyB but also of their conversions in membrane pore formation [12].

Binding to natural and artificial lipid membranes followed by permeabilisation is a common feature of many aegerolysins [13]. In line with this, OlyA/PlyB has been reported to induce erythrocyte lysis and to be cytotoxic for various cell lines at sub-micromolar concentrations [16]. The lytic process starts by recognition of distinctive raft-like membrane microdomains enriched in cholesterol and sphingomyelin [6,17]. Due to their important functions in living cells and cell-cell interactions [18,19,20], there is an increasing need for new techniques to study lipid rafts. OlyA/PlyB, which recognises specifically the combination of the two main lipid raft components, sphingomyelin and cholesterol, is in this regard a very good candidate for a new marker of raft-like membrane microdomains [21].

### 2.3. Main Pathophysiological Effects

Sporadic acute intoxications have been described in humans and animals after the ingestion of large quantities of fresh mushrooms [22]. Experimentally, OlyA/PlyB was shown to be toxic and lethal to rodents, with an estimated LD_50_ of 1170 µg/kg when administered by intravenous (i.v.) way [23]. Following i.v. administration of 1 mouse LD_50_, scratching, jumping, respiratory distress, cyanosis, paralysis and death of experimental mice generally occur within 3–5 min. After i.v. administration of 1 mouse LD_50_ into rats, OlyA/PlyB produced bradycardia, myocardial ischemia and ventricular extrasystoles accompanied by progressive fall of arterial blood pressure to the mid-circulatory pressure, leading to death of experimental animals [23]. Similar arrhythmias and time-course of arterial blood pressure produced by OlyA/PlyB were observed in pharmacologically vagotomised and artificially ventilated rats, indicating that vagotomy and hypoxia due to respiratory arrest after administration of this toxin are not primarily responsible for its cardiotoxicity [23]. OlyA/PlyB is lytic to human, bovine, sheep and rat erythrocytes at nanomolar range concentrations [23,24]. The mechanism responsible for the haemolytic effect of OlyA/PlyB is colloid-osmotic shock due to pore formation in cell membranes [24]. In addition, this toxin induces lysis of rat erythrocytes in vivo, as indicated by a significant increase in potassium concentration in serum (higher than 10 mM) and the red-coloured appearance of blood serum due to release of haemoglobin from damaged erythrocytes [23]. This observation was later confirmed by measuring the haemolytic activity on rat erythrocytes using a turbidimetric method [23]. Since such high blood potassium concentrations may cause cardiac arrest [25,26,27], it was concluded that hyperkalaemia probably plays an important role in the cardiotoxicity of OlyA/PlyB.

Respiratory arrest develops within few seconds after OlyA/PlyB administration, and hypoxia is a well-known cause of severe arrhythmias. However, arrhythmias also developed in artificially ventilated animals after i.v. administration of OlyA/PlyB, suggesting that mechanisms other than respiratory arrest are responsible for the cardiotoxic effects [23]. Myocardial hypoxia and cardiac arrest may be produced by sudden and significantly reduced blood flow through the coronary arteries. Since OlyA/PlyB is able to form pores in biological membranes [24], direct effects on porcine coronary arteries were studied. OlyA/PlyB induced a dose-dependent contraction of porcine coronary artery rings, as well as of rat aortas [28], and prevented the endothelium-mediated relaxation [29]. Contractile effects of OlyA/PlyB are probably due to direct effects on pig coronary smooth muscle cells. As revealed by fluorometric measurements, OlyA/PlyB increases the intracellular calcium concentration ([Ca^2+^]_i_) in smooth muscle A10 and NG108-15 cells, and alter their morphology which underlay its cardio- and neuro-toxic effects [30,31]. These effects can cause coronary vasoconstriction leading to myocardial ischemia accompanied by arrhythmias and heart failure. Pathological examination of main body tissues revealed that OlyA/PlyB induced perivascular oedema in the heart and lungs, as well as focal myocardial haemorrhages in rats injected with 1 mouse LD_50_. The mechanism underlying oedema and myocardial haemorrhages is a damage of endothelial cells, both in vitro and in vivo, as revealed by histological examination of tissues [29].

The biochemical properties and biological effects of OlyA/PlyB are summarised in Table 1.

### 2.4. Biological Use

OlyA/PlyB binds with high affinity to cholesterol and cholesterol/sphingomyelin-rich membrane domains in human urothelial cancer cells, and produces necrotic cell death [32]. Selectivity of OlyA/PlyB is based on the cholesterol and cholesterol/sphingomyelin-rich membrane domains in urothelial cancer cells, in contrast to normal urothelial cells. Fluorescent-labelled OlyA-mCherry has been utilised as a highly specific probe to visualise cholesterol/sphingomyelin rich membrane microdomains in living and fixed cells, and to study membrane trafficking [33].

## 3. Equinatoxins

### 3.1. Origin

Isolation of equinatoxin (EqT), a lethal protein from *Actinia equina L.*, was first described by Ferlan and Lebez [34]. Then, in 1988, isolation of three isotoxins (EqT I, EqT II and EqT III) was reported with mouse LD_50_ of 23, 35 and 83 µg/kg, respectively [35].

### 3.2. Chemical Structure and Biological Properties

EqTs are cytolytic water-soluble proteins that readily interact with cell and artificial lipid membranes containing sphingomyelin [35]. It has been shown that, in bovine lactotrophs, EqT II induces a rapid increase in [Ca^2+^]_i_ by the formation of Ca^2+^ permeable ion channels in lipid bilayers [37]. The composition could be resolved only after elucidation of the crystal structure of EqT II [3]. This was the first resolved structure of a eukaryote pore-forming protein. EqT II is a single-domain protein based on a 12 strand β-sandwich fold with a hydrophobic core and a pair of α-helices, each of which is associated with the face of a β-sheet. Initial experiments have shown that, in biological and artificial membranes, three to four EqT II molecules oligomerize and create cation-selective pores [38]. A detailed mechanism of pore formation by EqTs was described and reviewed by Anderluh et al. [39,40]. The high affinity of EqT II for membrane sphingomyelin makes it a suitable molecule for sensing membrane microdomains [21] and for membrane reorganization [41]. A neutron reflection study reveals new insight related to the binding process of EqT II into the plasma membrane. Thus, it was reported that EqT II binds in several distinct orientations which depend on membrane lipid composition [42]. Interestingly, EqT II exists on the cell surface as a mixture of oligomeric species including monomers, dimers, tetramers and hexamers [43]. Re-evaluation of the crystal structure of EqT II and other actinoporins strongly suggests that the pore formed by these toxins is composed of a matrix of alternating lipid and protein components and that octameric symmetry of the toxin structure in the pore is more probable than the previously suggested tetrameric symmetry [44,45]. Similarly, recent data based on experiments with fragaceatoxin suggest also that the toxin-lipid complexes in biological membranes are composed of two, four, six or eight subunits of toxin molecules. In particular, the pore formed by fragaceatoxin C is composed of eight toxin units alternating with pore-specific membrane lipids filling the gaps between the toxin units. [46].

### 3.3. Main Pathophysiological Effects

Cardiorespiratory arrest in rats caused by EqT was first described by Sket et al. [47] but, at that time, the underlying mechanism was not fully understood. In vitro studies revealed that EqT (80–200 ng/mL) increases the permeability and resistance of the lung vasculature and produces, at concentrations higher than 150 ng/mL, interstitial and alveolar pulmonary oedema [48]. At low concentrations (0.1–3 µg/mL), EqT induces a transient negative inotropic effect followed by a long-lasting positive inotropic effect in isolated guinea pig atrium. It was proposed that the formation of prostaglandin E2 is responsible for this effect, since it could be inhibited by indomethacin, a well-known inhibitor of prostaglandin synthesis [49]. The positive inotropic effect described by these authors was also seen in experiments performed on isolated guinea pig hearts using EqT II [50], but only when the toxin had been applied at low concentrations (picomolar range). Higher concentrations of EqT II caused a pronounced negative inotropic effect. Cardiorespiratory effects, similar to those produced by EqT [47], were later confirmed using EqT II [51] and EqT III [52]. EqT II causes negative inotropic and chronotropic effects such as bradycardia, action potential conduction disturbances and extrasystoles. Similarly, the lethal dose of EqT III also produces arrhythmias, a drop of arterial blood pressure and cardiac arrest. The two isotoxins are haemolytic, and it is well known that the hyperkalaemia caused by the lysis of erythrocytes can produce serious arrhythmias leading to cardiac failure. A detailed study of the role of haemolysis in EqT lethality revealed an only marginal role of hyperkalaemia in the cardiotoxic effects of these toxins [52]. This is in agreement with data showing that the more toxic EqT II is less haemolytic than EqT III. As EqT III is the least toxic but causes the most pronounced hyperkalaemia, it seems that the elevation of plasma K^+^ concentration is not the primary cause of cardiorespiratory arrest. In vivo, EqT II and EqT III cause similar alterations of electrocardiogram (ECG), breathing and blood pressure, indicating that the same cardiotoxicity mechanism may be involved.

EqTs belong to the group of pore forming toxins that enable passage of cations, mainly Ca^2+^, through phospholipid bilayers, including the plasma membrane. Therefore, another possible pathophysiological mechanism of the cardiotoxicity may be a decreased coronary perfusion due to vasoconstriction. Vasoconstrictor effects of EqT II may also include endothelin-dependent pathway [53]. EqT II-triggered endothelin release is probably one of the mechanisms involved in the lowering of coronary flow induced by this toxin [54], since endothelin is well known to activate L-type calcium channels in smooth muscle cells. As EqTs form cation selective pores in cellular membranes, a direct effect on smooth muscle cells in the vascular wall may also play a major role. To answer this question, porcine coronary arteries were exposed to EqT III (1–100 nM), and the resting tension of smooth muscle as well as the maximum force of contractions were measured. The results revealed that EqT III also directly triggers contraction of isolated porcine coronary arteries at nanomolar concentrations. This mechanism probably explains most of the EqT III cardiotoxic effects [52]. On Langendorff rat heart preparations, EqT II (0.1–10 nM) was reported to cause arrhythmia as well as decreased coronary perfusion rate and left ventricular pressure in a dose-dependent manner. At higher concentrations, EqT II produces cardiac arrest within a few seconds. As in the rat heart, EqT II also decreases coronary flow in the porcine heart. This effect could be abolished by an antagonist of L-type voltage-dependent calcium channels (i.e., Ca_V_1.2 channels) such as nicardipine. Additionally, it was shown that EqT II increases the tension of spontaneous contractions and induced long-lasting contracture of guinea pig *taenia caeci* smooth muscle, accompanied by a marked increase in [Ca^2+^]_i_ [55]. After i.v. administration, EqT II first enters into the right atrium and then the right ventricle of the heart before reaching the pulmonary circulation. EqTs have high binding affinity for sphingomyelin-rich cell membranes [5,56,57,58] and thus rapidly bind to blood cells and endothelium. In order to assess the possibility that a sufficient concentration of unbound EqT II is still present in the arterial blood and in the systemic circulation to produce direct cardiotoxic effects, perfusion experiments were performed on isolated rat lungs. After in vitro perfusion of the lung with a solution containing 100 nM EqT II, the toxin concentration in perfusates ranged between 0.8 and 5 nM. Effluent from the lungs contained enough EqT II to produce cardiotoxic effects on isolated Langendorff heart, as described previously [51]. This is in accordance with the findings that the lethal effects of EqT II are mainly attributed to its vasoconstrictor effects and direct cardiotoxicity. The mechanism of EqT II-induced respiratory arrest is not yet sufficiently explained. After i.v. administration of EqT I, EqT II or EqT III, the respiratory activity stops within a few seconds. It was shown, at least for EqT I, that electrical stimulation of the phrenic nerve triggers normal diaphragm muscle contraction indicating that neuromuscular transmission and function are unaffected by the toxin. Because respiratory arrest causes cardiac hypoxia, the alterations in blood pressure and electrical activity, similar to those observed after administration of one mouse LD_50_ of EqTs, could cause cardiac hypoxia. However, experiments performed on artificially ventilated animals, showed that artificial ventilation did not prevent the changes in ECG and blood pressure. Therefore, hypoxia was not confirmed as a primary cause for cardiotoxicity. Respiratory arrest may also be caused by respiratory reflexes activated through J-receptors in lung parenchyma, which are strongly stimulated under pathophysiological conditions like pulmonary oedema. EqTs are relatively large molecules (with molecular masses of around 20 kDa) and, due to their size, it is unlikely that they could pass the brain-blood barrier unless endothelial damage gives access to the neurons of the respiratory centre in the *medulla oblongata*. Recent preliminary results have shown that EqT II causes swelling and lysis of endothelial cells, an effect that may give toxin access to neuronal cells. Direct effects of EqTs on the respiratory centre cannot be excluded since EqT II has been reported to produce swelling of differentiated neuroblastoma NG108-15 cells [59]. Moreover, axonal swelling at the node of Ranvier of myelinated nerve fibres has also been observed in vitro after application of EqT II [60].

The biochemical properties and biological effects of EqT II are summarised in Table 2.

### 3.4. Biological Use

Despite the fact that low concentrations of EqTs are lethal, several studies have been reported on their effects on cancer cells [65,66,67]. Furthermore, they have been tested as the toxic moiety of immunotoxins targeting at *Giardia duodenalis* [68]. Their use as molecular probes for sphingomyelin-rich membrane domains seems to be more promising [21,69]. Finally, EqT II has been shown to inhibit endocytosis [41] and to cause membrane reorganization [41,70]. Development of immunotoxins targeting specific cells may be the most promising future use of equinatoxins.

## 4. Conclusions

The results of in vivo and in vitro studies indicate that hyperkalaemia that appears after the cytolytic action of OlyA/PlyB on blood and other exposed cells is mainly responsible for its cardiotoxic action. Coronary constriction and possible direct effect of OlyA/PlyB on cardiac tissue are additional mechanisms of cardiotoxicity. Besides direct cardiotoxic effects, OlyA/PlyB also damages endothelial cells and causes interstitial and alveolar oedema in vivo. This, together with coronary spasm, further amplifies tissue hypoxia and probably contributes to the respiratory arrest. All these mechanisms play an important role in the cardiorespiratory toxicity of OlyA/PlyB. On the other hand, EqTs are haemolytic, and the most studied EqT II also produces hyperkalaemia, but its effects are faster and hyperkalaemia seems to have a smaller effect on cardiac function than the direct action of the toxin. Both toxins have proven to be good tools to study cell membrane function as they bind to specific membrane domains. As potent cytotoxic agents they may be useful for designing new therapeutic substances. EqT II has already been considered as the toxic moiety of a potent immunotoxin.

## Figures and Tables

**Table 1:** Biochemical properties and biological effects of ostreolysin A/pleurotolysin B (OlyA/PlyB).

Biochemical Properties and Biological Effects		Reference
**Biochemical Properties**		
Molecular mass (kDa)	15 (OlyA)–59 (PlyB) molar ratio 9:1 (*Pleurotus ostreatus*)	[11,36]
pI	5.0	[7]
Molecular targets	Cholesterol and sphingomyelin	[6]
Activity	Pore formation	[2,24,36]
	Involvement in fructification of oyster mushroom	[7,15]
**Effects In Vivo**		
LD_50_ (µg/kg mouse)	1170	[23]
Circulation, heart (rat)	Progressive drop of arterial blood pressure	[23]
	Bradycardia, myocardial ischaemia	
	Ventricular extrasystoles	
Blood (rat)	Haemolysis Hyperkalaemia ([K^+^] > 10 mM)	[23]
**Effects on Isolated Organs**		
Rat aorta	Increase in aortic ring tension	[28]
Porcine coronary artery		[29]
**Cellular and Subcellular Effects**		
Human, bovine, sheep erythrocytes	Haemolysis (64 nM Oly)	[24]
Human umbilical vein endothelial cells	Toxicity (ED_50_ = 2.2 µg/mL Oly)	[28]
Chinese hamster lung fibroblasts	Toxicity (ED_50_ = 1.3 µg/mL Oly)	
A10 smooth muscle cells	Increase in [Ca^2+^]_i_ (≥14 nM OlyA/1.56 nM PlyB)	[31]
NG 108-15 cells	Increase in [Ca^2+^]_i_ (≥7 nM OlyA/0.78 nM PlyB)	[30]
	Cell swelling, plasma membrane blebbing (≥700 nM OlyA /78 nM PlyB)	

**Table 2:** Biochemical properties and biological effects of equinatoxin (EqT) II.

Biochemical Properties and Biological Effects		Reference
**Biochemical Properties**		
Molecular mass (kDa)	20	[35]
pI	10.5	[35]
Molecular targets	Sphingomyelin	[38]
Activity	Pore formation	[37,38]
**Effects In Vivo**		
LD_50_ (µg/kg mouse)	35	[35]
Circulation, heart (rat)	Bradycardia, hypotension, extrasystoles	[61]
Blood (rat)	Platelet aggregation	[61]
Respiration (rat)	Respiratory arrest	[47]
**Effects on Isolated Organs**		
Rat skeletal muscle	Spontaneous twitches (10 nM)	[62]
Rat heart (Langendorff preparation)	Drop in perfusion rate, decreased left ventricular pressure, arrhythmia (0.1–10 nM)	[51]
Porcine coronary artery	Vasoconstriction (EC_50_ = 101.1 nM)	[53]
**Cellular and Subcellular Effects**		
Human erythrocytes	Haemolysis	[38]
	Pore formation/*r* = 1.1 nm (50 ng/mL)	
Rabbit Platelets	Aggregation (0.01 ng/mL)	[63]
V-79-379 A cell line	Toxicity (ED_50_ = 17 ng/mL)	[64]
Bovine lactotrophs Planar lipid bilayers	Toxicity (230 nM) Ion channel formation (650 nM) Increase in [Ca^2+^]_i_ (230 nM)	[37]
NG 108-15 cells	Cell swelling, increase in [Ca^2+^]_i_	[59]
ECV-304 cells	Cell swelling, lysis (1–10 nM)	[49]
*Taenia caeci* smooth muscle cells	Increase in [Ca^2+^]_i_, muscle contraction (10–500 nM)	[55]

## References

[1] Gouaux E. (1997). Channel-forming toxins: Tales of transformation. Curr. Opin. Struct. Biol..

[2] Ota K., Leonardi A., Mikelj M., Skočaj M., Wohlschlager T., Kunzler M., Aebi M., Narat M., Križaj I., Anderluh G. (2013). Membrane cholesterol and sphingomyelin, and ostreolysin a are obligatory for pore-formation by a macpf/cdc-like pore-forming protein, pleurotolysin b. Biochimie.

[3] Athanasiadis A., Anderluh G., Maček P., Turk D. (2001). Crystal structure of the soluble form of equinatoxin II, a pore-forming toxin from the sea anemone *Actinia equina*. Structure.

[4] Berne S., Sepčić K., Anderluh G., Turk T., Maček P., Poklar Ulrih N. (2005). Effect of pH on the pore forming activity and conformational stability of ostreolysin, a lipid raft-binding protein from the edible mushroom *Pleurotus ostreatus*. Biochemistry.

[5] Bakrač B., Gutierrez-Aguirre I., Podlesek Z., Sonnen A.F., Gilbert R.J., Maček P., Lakey J.H., Anderluh G. (2008). Molecular determinants of sphingomyelin specificity of a eukaryotic pore-forming toxin. J. Biol. Chem..

[6] Sepčić K., Berne S., Rebolj K., Batista U., Plemenitaš A., Šentjurc M., Maček P. (2004). Ostreolysin, a pore-forming protein from the oyster mushroom, interacts specifically with membrane cholesterol-rich lipid domains. FEBS Lett..

[7] Berne S., Križaj I., Pohleven F., Turk T., Maček P., Sepčić K. (2002). Pleurotus and agrocybe hemolysins, new proteins hypothetically involved in fungal fruiting. Biochim.Biophys. Acta.

[8] Fernandez Espinar M.T., Labarere J. (1997). Cloning and sequencing of the aa-pri1 gene specifically expressed during fruiting initiation in the edible mushroom *Agrocybe aegerita*, and analysis of the predicted amino-acid sequence. Curr. Genet..

[9] Ebina K., Sakagami H., Yokota K., Kondo H. (1994). Cloning and nucleotide sequence of cdna encoding asp-hemolysin from *Aspergillus fumigatus*. Biochim. Biophys. Acta.

[10] Barloy F., Lecadet M.M., Delecluse A. (1998). Cloning and sequencing of three new putative toxin genes from *Clostridium bifermentans* CH18. Gene.

[11] Ota K., Butala M., Viero G., Dalla Serra M., Sepčić K., Maček P. (2014). Fungal macpf-like proteins and aegerolysins: Bi-component pore-forming proteins?. Subcell. Biochem..

[12] Lukoyanova N., Kondos S.C., Farabella I., Law R.H.P., Reboul C.F., Caradoc-Davies T.T., Spicer B.A., Kleifeld O., Traore D.A., Ekkel S.M. (2015). Conformational changes during pore formation by the perforin-related protein pleurotolysin. PLoS Biol..

[13] Berne S., Lah L., Sepčić K. (2009). Aegerolysins: Structure, function, and putative biological role. Protein Sci. Publ. Protein Soc..

[14] Nayak A.P., Green B.J., Beezhold D.H. (2013). Fungal hemolysins. Med. Mycol..

[15] Vidic I., Berne S., Drobne D., Maček P., Frangež R., Turk T., Štrus J., Sepčić K. (2005). Temporal and spatial expression of ostreolysin during development of the oyster mushroom (*Pleurotus ostreatus*). Mycol. Res..

[16] Sepčić K., Frangež R., Gupta V. (2010). Cytolytic and toxic effects of ostreolysin, a protein from the oyster mushroom (*Pleurotus ostreatus*). Comprehensive Bioactive Natural Products.

[17] Chowdhury H.H., Rebolj K., Kreft M., Zorec R., Maček P., Sepčić K. (2008). Lysophospholipids prevent binding of a cytolytic protein ostreolysin to cholesterol-enriched membrane domains. Toxicon.

[18] Edidin M. (2003). The state of lipid rafts: From model membranes to cells. Annu. Rev. Biophys. Biomol. Struct..

[19] London E. (2002). Insights into lipid raft structure and formation from experiments in model membranes. Curr. Opin. Struct. Biol..

[20] Simons K., Ikonen E. (1997). Functional rafts in cell membranes. Nature.

[21] Skočaj M., Bakrač B., Križaj I., Maček P., Anderluh G., Sepčić K. (2013). The sensing of membrane microdomains based on pore-forming toxins. Curr. Med. Chem..

[22] Al-Deen I.H., Twaij H.A., Al-Badr A.A., Istarabadi T.A. (1987). Toxicologic and histopathologic studies of *Pleurotus ostreatus* mushroom in mice. J. Ethnopharmacol..

[23] Žužek M.C., Maček P., Sepčić K., Cestnik V., Frangež R. (2006). Toxic and lethal effects of ostreolysin, a cytolytic protein from edible oyster mushroom (*Pleurotus ostreatus*), in rodents. Toxicon.

[24] Sepčić K., Berne S., Potrich C., Turk T., Maček P., Menestrina G. (2003). Interaction of ostreolysin, a cytolytic protein from the edible mushroom *Pleurotus ostreatus*, with lipid membranes and modulation by lysophospholipids. Eur. J. Biochem. FEBS.

[25] Emberson J.W., Muir A.R. (1969). Changes in the ultrastructure of rat myocardium induced by hyperkalaemia. J. Anat..

[26] Parham W.A., Mehdirad A.A., Biermann K.M., Fredman C.S. (2006). Hyperkalemia revisited. Tex. Heart Inst. J..

[27] Van der Meer C., Valkenburg P.W., Snijders P.M. (1986). Studies on hyperkalemia as a cause of death in intestinal ischemia shock in rats. Circ. Shock.

[28] Rebolj K., Batista U., Sepčić K., Cestnik V., Maček P., Frangež R. (2007). Ostreolysin affects rat aorta ring tension and endothelial cell viability in vitro. Toxicon.

[29] Juntes P., Rebolj K., Sepčić K., Maček P., Žužek M.C., Cestnik V., Frangež R. (2009). Ostreolysin induces sustained contraction of porcine coronary arteries and endothelial dysfunction in middle- and large-sized vessels. Toxicon.

[30] Vrecl M., Babnik M., Diacci U., Benoit E., Frangež R. (2015). Effect of the ostreolysin a/pleurotolysin b pore-forming complex on neuroblastoma cell morphology and intracellular Ca^2+^ activity. Toxicol. Sci..

[31] Vrecl M., Babnik M., Sepčić K., Žužek M.C., Maček P., Diacci U., Frangež R. (2015). Effect of the ostreolysin a/pleurotolysin b pore-forming complex on intracellular Ca^2+^ activity in the vascular smooth muscle cell line A10. Toxicol. In Vitro.

[32] Resnik N., Repnik U., Kreft M.E., Sepčić K., Maček P., Turk B., Veranič P. (2015). Highly selective anti-cancer activity of cholesterol-interacting agents methyl-beta-cyclodextrin and ostreolysin a/pleurotolysin b protein complex on urothelial cancer cells. PLoS ONE.

[33] Skočaj M., Resnik N., Grundner M., Ota K., Rojko N., Hodnik V., Anderluh G., Sobota A., Maček P., Veranič P. (2014). Tracking cholesterol/sphingomyelin-rich membrane domains with the ostreolysin a-mcherry protein. PLoS ONE.

[34] Ferlan I., Lebez D. (1974). Equinatoxin, a lethal protein from *Actinia equina* L. Purification and characterization. Toxicon.

[35] Maček P., Lebez D. (1988). Isolation and characterization of three lethal and hemolytic toxins from the sea anemone *Actinia equina* L.. Toxicon.

[36] Schlumberger S., Kristan K.C., Ota K., Frangež R., Molgó J., Sepčić K., Benoit E., Maček P. (2014). Permeability characteristics of cell-membrane pores induced by ostreolysin a/pleurotolysin b, binary pore-forming proteins from the oyster mushroom. FEBS Lett..

[37] Zorec R., Tester M., Maček P., Mason W.T. (1990). Cytotoxicity of equinatoxin II from the sea anemone *Actinia equina* involves ion channel formation and an increase in intracellular calcium activity. J. Membr. Biol..

[38] Belmonte G., Pederzolli C., Maček P., Menestrina G. (1993). Pore formation by the sea anemone cytolysin equinatoxin II in red blood cells and model lipid membranes. J. Membr. Biol..

[39] Anderluh G., Dalla Serra M., Viero G., Guella G., Maček P., Menestrina G. (2003). Pore formation by equinatoxin II, a eukaryotic protein toxin, occurs by induction of nonlamellar lipid structures. J. Biol. Chem..

[40] Anderluh G., Maček P., Lakey J.H. (2003). Peeking into a secret world of pore-forming toxins: Membrane binding processes studied by surface plasmon resonance. Toxicon.

[41] Garcia-Saez A.J., Buschhorn S.B., Keller H., Anderluh G., Simons K., Schwille P. (2011). Oligomerization and pore formation by equinatoxin II inhibit endocytosis and lead to plasma membrane reorganization. J. Biol. Chem..

[42] Wacklin H.P., Bremec B.B., Moulin M., Rojko N., Haertlein M., Forsyth T., Anderluh G., Norton R.S. (2016). Neutron reflection study of the interaction of the eukaryotic pore-forming actinoporin equinatoxin II with lipid membranes reveals intermediate states in pore formation. Biochim. Biophys. Acta.

[43] Subburaj Y., Ros U., Hermann E., Tong R., Garcia-Saez A.J. (2015). Toxicity of an alpha-pore-forming toxin depends on the assembly mechanism on the target membrane as revealed by single molecule imaging. J. Biol. Chem..

[44] Mancheno J.M., Martin-Benito J., Martinez-Ripoll M., Gavilanes J.G., Hermoso J.A. (2003). Crystal and electron microscopy structures of sticholysin II actinoporin reveal insights, into the mechanism of membrane pore formation. Structure.

[45] Gilbert R.J.C. (2016). Protein–lipid interactions and non-lamellar lipidic structures in membrane pore formation and membrane fusion. Biochim. Biophys. Acta.

[46] Tanaka K., Caaveiro J.M., Morante K., Gonzalez-Manas J.M., Tsumoto K. (2015). Structural basis for self-assembly of a cytolytic pore lined by protein and lipid. Nat. Commun,.

[47] Sket D., Drašlar K., Ferlan I., Lebez D. (1974). Equinatoxin, a lethal protein from *Actinia equina*. II. Pathophysiological action. Toxicon.

[48] Lafranconi W.M., Ferlan I., Russell F.E., Huxtable R.J. (1984). The action of equinatoxin, a peptide from the venom of the sea anemone, *Actinia equina*, on the isolated lung. Toxicon.

[49] Ho C.L., Ko J.L., Lue H.M., Lee C.Y., Ferlan I. (1987). Effects of equinatoxin on the guinea-pig atrium. Toxicon.

[50] Budihna M., Maček P., Šuput D. (1990). Effects of equinatoxin II on the isolated guinea-pig heart. Eur. J. Pharmacol..

[51] Bunc M., Drevenšek G., Budihna M., Šuput D. (1999). Effects of equinatoxin II from *Actinia equina* (L.) on isolated rat heart: The role of direct cardiotoxic effects in equinatoxin II lethality. Toxicon.

[52] Šuput D., Frangež R., Bunc M. (2001). Cardiovascular effects of equinatoxin III from the sea anemone *Actinia equina* (L.). Toxicon.

[53] Drevenšek G., Kirbiš S., Bunc M., Žitko M., Budihna M.V., Šuput D. (2002). Tezosentan inhibits both equinatoxin II and endotelin-1 induced contractions of isolated porcine coronary artery in a similar way. J. Nat. Toxins.

[54] Bunc M., Rozman J., Vidmar A., Rakovec P., Drevenšek G., Budihna M.V., Šuput D. (2002). Nicardipine diminished equinatoxin II-induced decrease of coronary flow in isolated rat and pig hearts. Cell. Mol. Biol. Lett..

[55] Frangež R., Šuput D., Molgó J. (2008). Effects of equinatoxin II on isolated guinea pig *taenia caeci* muscle contractility and intracellular Ca^2+^. Toxicon.

[56] Belmonte G., Menestrina G., Pederzolli C., Križaj I., Gubenšek F., Turk T., Maček P. (1994). Primary and secondary structure of a pore-forming toxin from the sea anemone, *Actinia equina* L., and its association with lipid vesicles. Biochim. Biophys. Acta.

[57] Maček P., Belmonte G., Pederzolli C., Menestrina G. (1994). Mechanism of action of equinatoxin II, a cytolysin from the sea anemone *Actinia equina* L. belonging to the family of actinoporins. Toxicology.

[58] Maček P., Zecchini M., Pederzolli C., Dalla Serra M., Menestrina G. (1995). Intrinsic tryptophan fluorescence of equinatoxin II, a pore-forming polypeptide from the sea anemone *Actinia equina* L, monitors its interaction with lipid membranes. Eur. J. Biochem. FEBS.

[59] Meunier F.A., Frangež R., Benoit E., Ouanounou G., Rouzaire-Dubois B., Šuput D., Molgó J. (2000). Ca(2+) and Na(+) contribute to the swelling of differentiated neuroblastoma cells induced by equinatoxin-II. Toxicon.

[60] Benoit E., Mattei C., Ouanounou G., Meunier F.A., Šuput D., Le Gall F., Marquais M., Dechraoui M.Y., Molgó J. (2002). Ionic mechanisms involved in the nodal swelling of myelinated axons caused by marine toxins. Cell. Mol. Biol. Lett..

[61] Bunc M., Frangež R., Horvat I., Turk T., Šuput D. (1994). Effects of equinatoxins in vivo. Possible role of degranulation of thrombocytes and granulocytes. Ann. N. Y. Acad. Sci..

[62] Horvat-Znidaršič I., Šuput D. (1996). The effect of equinatoxin II on nerve and muscle. Pflug. Arch. Eur. J. Physiol..

[63] Teng C.M., Lee L.G., Lee C.Y., Ferlan I. (1988). Platelet aggregation induced by equinatoxin. Thromb. Res..

[64] Batista U., Maček P., Sedmak B. (1990). The cytotoxic and cytolytic activity of equinatoxin II from the sea anemone *Actinia equina*. Cell Biol. Int. Rep..

[65] Giraldi T., Ferlan I., Romeo D. (1976). Antitumor activity of equinatoxin. Chem. Biol. Interact..

[66] Kahn S.A., Biasoli D., Garcia C., Geraldo L.H., Pontes B., Sobrinho M., Frauches A.C., Romao L., Soletti R.C., Assuncao Fdos S. (2012). Equinatoxin II potentiates temozolomide- and etoposide-induced glioblastoma cell death. Curr. Top. Med. Chem..

[67] Soletti R.C., de Faria G.P., Vernal J., Terenzi H., Anderluh G., Borges H.L., Moura-Neto V., Gabilan N.H. (2008). Potentiation of anticancer-drug cytotoxicity by sea anemone pore-forming proteins in human glioblastoma cells. Anticancer Drugs.

[68] Tejuca M., Anderluh G., Maček P., Marcet R., Torres D., Sarracent J., Alvarez C., Lanio M.E., Dalla Serra M., Menestrina G. (1999). Antiparasite activity of sea-anemone cytolysins on *Giardia duodenalis* and specific targeting with anti-giardia antibodies. Int. J. Parasitol..

[69] Yamaji-Hasegawa A., Hullin-Matsuda F., Greimel P., Kobayashi T. (2016). Pore-forming toxins: Properties, diversity, and uses as tools to image sphingomyelin and ceramide phosphoethanolamine. Biochim. Biophys. Acta.

[70] Šentjurc M., Štalc A., Šuput D. (1996). Influence of equinatoxin II on coronary smooth muscle membrane fluidity. Pflug. Arch. Eur. J. Physiol..

